# An edge-driven multi-agent optimization model for infectious disease detection

**DOI:** 10.1007/s10489-021-03145-0

**Published:** 2022-03-07

**Authors:** Youcef Djenouri, Gautam Srivastava, Anis Yazidi, Jerry Chun-Wei Lin

**Affiliations:** 1 SINTEF Digital, Oslo, Norway; 2 Brandon University, Brandon, Canada; 3 China Medical University, Taichung, Taiwan; 4 Oslomet, Oslo, Norway; 5 Western Norway University of Applied Sciences, Bergen, Norway

**Keywords:** Edge-Driven, Infection disease, Multi-agents system, Evolutionary computation

## Abstract

This research work introduces a new intelligent framework for infectious disease detection by exploring various emerging and intelligent paradigms. We propose new deep learning architectures such as entity embedding networks, long-short term memory, and convolution neural networks, for accurately learning heterogeneous medical data in identifying disease infection. The multi-agent system is also consolidated for increasing the autonomy behaviours of the proposed framework, where each agent can easily share the derived learning outputs with the other agents in the system. Furthermore, evolutionary computation algorithms, such as memetic algorithms, and bee swarm optimization controlled the exploration of the hyper-optimization parameter space of the proposed framework. Intensive experimentation has been established on medical data. Strong results obtained confirm the superiority of our framework against the solutions that are state of the art, in both detection rate, and runtime performance, where the detection rate reaches 98% for handling real use cases.

## Introduction

Technologies for controlling, managing, and detecting infectious diseases have shown potential interest in the last two years, in particular since the beginning of the COVID-19 pandemic [11, 12]. This gives rise to the need to design and create new intelligent systems for infection disease management in a medical setting environment. Infectious disease intelligence is becoming an interesting topic, in particular by taking advantage of emerging topics in artificial intelligence [4, 23]. When looking into multi-agent systems, deep learning networks, as well as evolutionary computation, these artificial intelligence-based technologies are the most used in medical applications [24, 29].

### Motivations

Deep learning is the field of artificial intelligence, which consists of complex neural architectures with a high number of layers and parameters to be tuned. These architectures are used not only for learning but also capable to extract relevant features directly from massive amounts of data. An exciting topic within deep learning is that of the analysis of medical data, and in particular disease infections [32, 33]. For instance, Wang et al. [32] developed a smart model for estimating the infection rate from COVID-19 samples. It combines both supervised and unsupervised learning strategies to achieve a $40\%$ improvement of detection speed. Wang et al. [33] analyzed pathogen images confirmed COVID-19 cases with the typical virus-based pneumonia while making use of learning through transfer (transfer learning). The result shows the great benefits obtained of using intelligent methods for COVID-19 diagnosis. In the novel realm of what is called distributed deep learning, we can also see examples that are largely investigated by exploring different types of DL models in well-established medical environments [3, 37]. The overall goal of the deep learning solutions, including distributed ones, is to identify disease infections to guide the medical staff in reaching fair and reasonable decisions in diagnosing different diseases. The disease infection identification tolerates various limitations, in particular the data heterogeneity where medical data may be structured such as row data, unstructured such as time series and/or complex data such as images data. This data diversity led the learning process untrustworthy. To eliminate these drawbacks, our work here is to investigate intelligent end-to-end solutions that require the use of deep learning (DL) as well as multi-agent systems (MAS).

Another important issue of existing infectious disease solutions is the huge number of hyper-parameters provided by the deep learning models. Randomly choosing these parameters, drastically decreased the performance in the entire learning process. Besides, the parameter setting procedure of such kind of framework is high time-consuming without any guarantee to reach reasonable convergence. Motivated by the success of evolutionary computation (EC) in solving complex problems [1], this research work incorporates the evolutionary computation to tune the parameters of the HI2D framework.

### Contributions

This research work is the initial work that will explore an in-depth combination of multi-agent systems, evolutionary computation as well as deep learning for disease detection. The main contributions can be provided in the following list:

1. HI2D (Hybrid Intelligence Infectious Disease), a novel new framework, is presented which adopts DL, MAS, and EC for the infectious disease identification. Each agent applies different deep learning architectures to learn from the medical training data, the different infectious diseases. The communication among the different agents is established at each iteration of the framework for knowledge sharing, reduction of the error learning rate.
2. We present a new adaptation of different deep learning models for handling heterogeneous data (structured, unstructured, and/or complex). The convolution NN, the LSTM, as well as entity-embedding network architectures are developed for handling row data, images, and time-series data in a medical setting. An adaptation of the deep learning models is ensured by different optimizations such as batch normalization, and dropout mechanisms.
3. We proposed new evolutionary computation algorithms based on the memetic algorithm, and the bees swarm optimization for intelligently exploring the configuration space of the different hyper-parameters values. This hyper-parameters optimization procedure allows better convergence of the proposed framework in learning the infection disease medical data.
4. Extensive experiments were carried out for showing the applicability of the HI2D framework. Our in-depth experimental results indicate that the HI2D outperformed other well-known infectious disease detection algorithms in terms of detection accuracy and runtime simultaneously.

### Paper outline

Paper organization is then shown as follows. Section 2 gives an in depth related work study in infectious disease technologies. Section 3 then presents a detailed explanation of the HI2D methodology. A performance evaluation of the HI2D framework is provided in Section 4. Section 5 discusses the main outcomes of applying the proposed framework on medical data, and the possible future directions of this research work. To conclude, Section 6 ends the paper.

## Related works: A literature review

Here, the main body of the related literature is briefly discussed in deep medical learning and infectious disease intelligence.

### Deep medical learning

Nicolau et al. [22] evaluated and reviewed a variety of solutions that are both interactive as well as medical learning-based in the domain of surgical oncology, highlighting each system’s strengths and weaknesses. Additionally, it discusses the potential use of computer vision (CV) techniques in the domain of surgical oncology. Chien et al. [36] devised an in-depth expanded alignment-based approach for use with data from image surgery. To create the head surface knowledge, the Red/Green/Blue-Depth data is utilized as well as point clouds. A group of Hololens captures alignment after it is optimized, allowing for simultaneous visualization of a surgical medical image as well as the head surface in a VR (virtual reality) environment. Sanchez et al. [9] created a methodology where medical images can be 3D reconstructed and visualized. It created fully automated end-2-end solutions to view medically gathered data using augmented and virtual reality. The process is completely automated and does not require medical team involvement. This specific project was tried on medical professionals for validation and solicit feedback on how to improve it.

Niu et al. [17] developed a deep understanding of adversarial attacks that could potentially occur on medical images and the ability to both detect and generate them. Medical training images that are used in the training of deep neural network classifiers were experimented with. A gradient operator is administered for the extraction of the perturbation features. To be able to identify and detect these perturbations, these features used in perturbation are included in the image test set. Muller et al. [20] gave an in-depth deep learning medical picture segmentation framework written in Python. Many data augmentation techniques were used, for example, colour augmentations, as well as spatial augmentation, were administered in the framework, as well as the well-known overly efficient U-Net algorithm that can be used exclusively in medical picture segmentation. Taghanaki et al. [31] conducted a review of existing solutions in the realm of image segmentation from medical sources and also created a novel taxonomy for existing solutions. Their taxonomy was able to encompass things at the level of architecture, as well as the type of user data, the type of loss functions that were used, sequence models that were used, and finally method’s level of supervision. Additionally, the taxonomy itself also included an in-depth examination of each algorithm’s category-created overall sub-groups for all algorithms within the taxonomy itself.

Gupta et al. [10] created a DL-based hierarchical multi-agent network that was able to successfully group all system-based end-user queries, then integrates them to solve the problem of answer prediction. In the domain of question answering visually, Gupta proposed a technique that segregated questions. The model for question answering is then integrated into the hierarchical deep multi-modal neural network that can predict answers. In addition, Chai et al. [7] developed a deep probabilistic model for representing uncertainty in the management of multiple medical data sources. After formulating the multi-source learning problem, Bayesian DL can be used for the extraction of uncertain features that could be potentially useful in the detection of glaucoma.

### Infectious disease intelligence

Hawaz et al. [21] developed an intelligent pattern mining-based method for identifying COVID diseases. The medical data are first transformed into the transactional database, where pattern mining is applied to study the correlation between different medical sequences. The prediction model is then trained to predict the behaviour of the new genome sequences. Lai et al. [32] proposed the use of intelligent models for automatic assessment of images to provide an informed estimation of the illusory COVID-19 infection rate. Both segmentation and classification tasks are considered, resulting in a 30 to 40 percent gain in detection time. Jain et al. [14] used three deep learning models to detect COVID-19 on chest radiographs. Data augmentation is first performed to enrich the training data and thus increase the learning performance. In addition, the deep architectures Inception V3, Xception, and ResNeXt are implemented for the use case of detecting COVID-19.

Chae et al. [6] investigated a new deep learning solution for infectious diseases. Two different models were proposed to deal with huge amounts of data: learning with long-term short-term memory and autoregressive integrated moving average. Moreover, an ensemble-based solution is integrated with contextual information obtained from social network analysis to enhance the performance of the proposed system. Wang et al. [33] analyzed 1, 065 pathogen images and confirmed COVID-19 cases with typical viral pneumonia using transfer learning. According to the result, it shows the benefit of using intelligent methods for COVID-19 diagnosis. Ahuja et al. [2] used four deep learning models ResNet18, ResNet50, ResNet101, and SqueezeNet to automatically identify COVID-19 from scan sections of the lungs CT. It used transfer learning in which the pre-trained models are considered in the learning phase.

Wong et al. [34] discussed the use of data management and artificial intelligence for data-driven infectious disease. This combination allows the healthcare services, and medical teams to reduce the risk of infection, and better for diagnosis. Hirano et al. [13] developed a model for infection disease classification using deep learning. It concentrated on three types of medical image classification: skin cancer classification using photographic images, referable diabetic retinopathy classification using X-ray chest images, and pneumonia classification using X-ray chest images. Transfer learning is used to derive the deep neural network models from various medical image diagnosis models. Additionally, the adversarial defence is considered by evaluating the deep neural network’s increased robustness to both non-targeted and targeted attacks. Jamshidi et al. [15] deal with heterogeneous medical data, both structured and unstructured data sources. It proposed the use of generative adversarial networks, extreme learning machines, and long-term memory to improve COVID-19 disease diagnosis and treatment.

Singh et al. [30] developed a deep learning model for disease and pest infection detection in a coconut tree. The images are first segmented using the *k*-means algorithm. The convolution neural network used the segmented images as input for disease prediction. Sedik et al. [27] combines the convolution neural network with the long short-term network and developed an intelligent deep learning solution for COVID-19 detection. They also contributed to collecting medical data and designed new medical data, which integrates the computed tomography and the X-ray images in normal and COVID-19 cases. Shalbaf et al. [28] used 15 pre-trained convolutional neural networks based on inception, ResNet, and DenseNet architectures to automatically identify the COVID-19. They also developed an ensemble model based on the first 15 models using the majority voting strategy for further boosting the identification process.

### Discussion

As can be seen from the above brief literature review, there has been a lot of research in the field of deep learning for medical applications. These methods explore learning mechanisms to create automated models for intelligent exploration the infectious disease. These techniques have a long way to go in the medical field, where such efforts are required in a variety of ways. In comparison, we propose the first dedicated complete framework in this paper that integrates deep learning, multi-agent systems, and evolutionary computation to achieve mature solutions for infection disease.

## HI2D: Hybrid intelligence infectious disease

### Principle

**Fig. 1:**
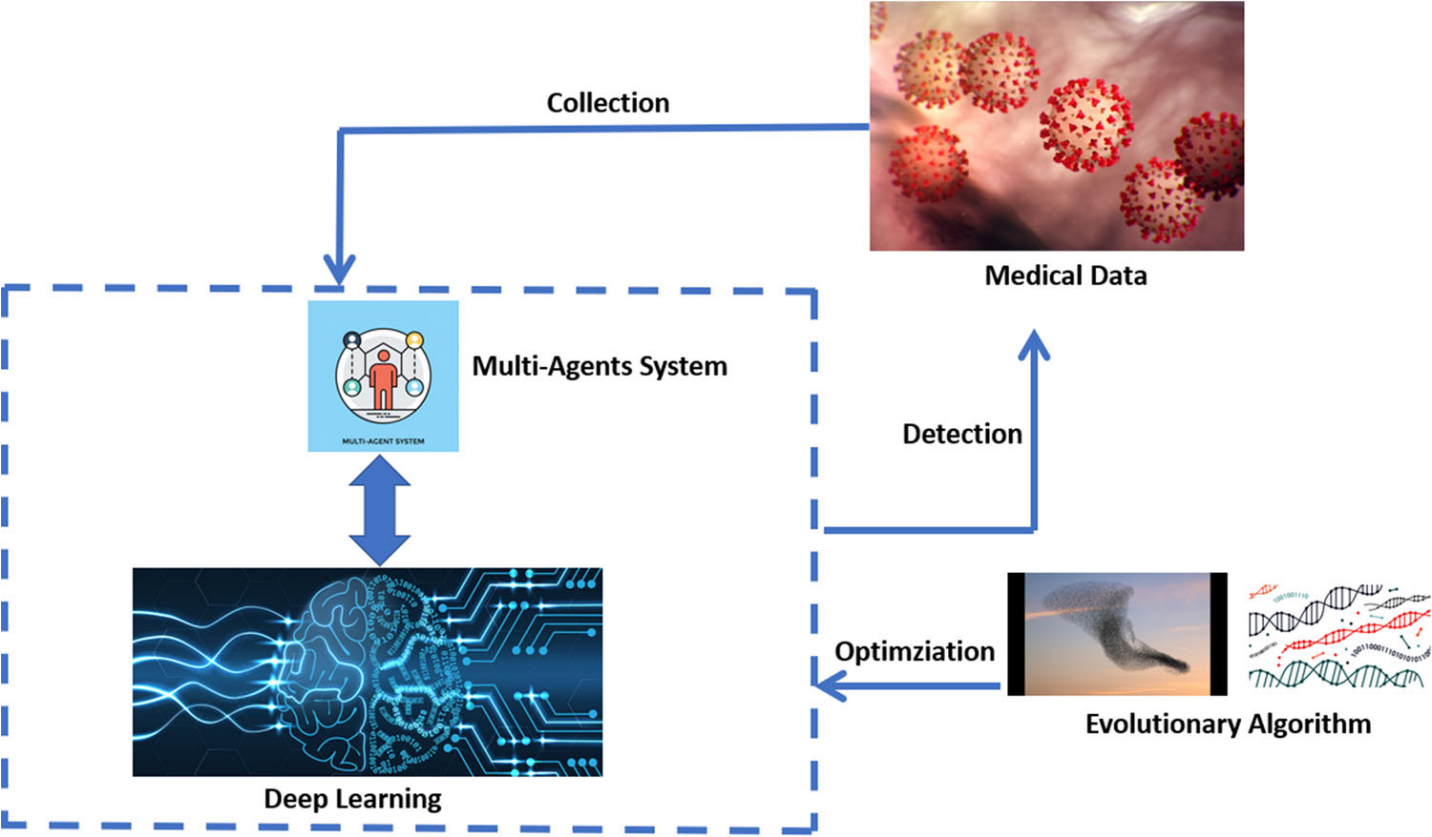
HI2D Framework

We begin here with the key elements of the HI2D (Hybrid Intelligence Infectious Disease). As shown in Fig. 1, HI2D is the combination of various smart methods for solving infectious disease challenges. Deep architectures such as long-term short memory (LSTM), Entity-Embedding Deep Learning (EEDL), and Convolution Neural Network (CNN) are adopted for handling the infectious disease. Heterogeneous medical data can be stored in different representations. LSTM is used to handle time-series data, EEDL is used to deal with structured and categorical data, and CNN is used for exploring medical image data. To accurately execute the HI2D in an edge-computing environment, the multi-agent system is investigated, where each agent can benefit from the environment by using the reinforcement learning paradigm. As deep learning provides a high number of parameters to be tuned, for some architectures it reaches million of parameters, the evolutionary computation is involved for finding the optimal parameters in real-time processing. In the next parts of this section, we focus on the components of H2ID.

### Deep learning

Different types of medical data can be collected from sensors in an edge-computing environment. In this research work, we explore three different deep learning architectures to learn from time series, categorical, structural, and image data.

1. **LSTM** [16]: Time series data is fed into this architecture. It is a collection of values from various periods. The network consists of many layers, each with a large number of neurons. In each layer, all neurons have the same weights, i.e., all neurons in the $i^{th}$ layer share the weights with the $i+1^{th}$. The input layer neurons are linked to the medical time series data such that each point value in the time series is associated with a single neuron in the input layer. The output data from this layer is weighted equally with that of the neurons in the final output layer. The target is to minimize the discrepancy between the output of the network and the actual data. To obtain $O^i_j$, we use the activation function on the $i^{th}$ neuron in the $j^{th}$ layer. When the outputs of all neurons in a layer are combined, a normalization method should be performed. First, the weight values of all neurons in the network are set to zero, and the process begins. Each time series of injected medical data is used to estimate the output data and determine the error. With each new layer of the network, the weights are recalculated. Once the entire set of medical time series data has been processed, the process is repeated.
2. **Entity-Embedding DL** [5]: An entity-embedded DL architecture is proposed for dealing with structured medical data. The structure is first represented in a vector of features using embeddings. Before being connected to the output layer, the feature vectors are connected to two other layers that are fully connected. We compressed the structured data to a feature vector using a bag of words solution. The number of visual words used to represent the data space is first calculated from the data. The intersection of the words and the data in each row is calculated, resulting in a matrix named *DW*. It consists of *d* rows, and *w* columns, in which *d* represents the number of all samples, and *w* represents the number of words. Each element (*i*, *j*) in *DW* shows the presence/absence of the $i^{th}$ data in the $j^{th}$ word.
3. **Convolution (NN) Neural Network** [19]: To deal with medical image data, we propose the use of a convolutional network architecture. In computer vision applications such as object identification and recognition, CNNs are a common type of deep architecture. Both time series and text data have benefited from the versatility of this technique in recent years. CNNs are based on the principle of using convolutional filters to extract features from matrix data. Convolutional filters use a set of weights on the matrix data of each pixel to create a new image. Back-propagation methods are used to reveal and fine-tune these weights.

Additionally, the batch normalization and dropout, well-known operators, for deep learning models are used in the training to increase the accuracy of the proposed framework. The batch normalization helps in faster converge of the network, where the Dropout is a regulator which helps avoid overfitting. Both mechanisms are crucial for the network to get good accuracy. The detailed description is as follows:

1. **Batch Normalization**: We used the batch normalization strategy in all deep networks proposed in this research work for efficiently training a high number of layers. This allows a better convergence of the learning process, with a few epochs. The batch normalization is performed after each convolution layer in CNN. It is applied to the hidden states of the LSTM network, and the Embedding-based network.
2. **Dropout**: It is a strategy to avoid over-fitting during the training process. It randomly skips the outputs of the neurons of the hidden layers at each wights updating phase. In the inference stage, it is straightforward to converge the predictions, by propagating a deep network with a small number of weights.

### Multi-agents systems

The tuple $<\mathcal {A},\mathcal {S},\mathcal {U},\mathcal {R}>$. $\mathcal {A}$ defines a multiagent system. There are $\mathcal {A}$ agents in total, and each of them is considered a separate Markov decision process in this context. There is a finite set of environmental states represented by $\mathcal {S}$, a set of actions represented by $\mathcal {U}$, and a reward function represented by $\mathcal {R}$. The strategies in $\mathcal {A}$ specify how each agent should behave given the current state and how it should make decisions about those actions. For example, in prediction, the goal of each agent is to find an optimal strategy that maximizes the specified objective function, e.g., the number of correctly predicted objects. The following sections detail the various components of our multi-agent system:

1. **Environment:** The environment is a collection of databases containing a massive amount of data from smart sensor devices. This enables the environment to generate specific states for the agent’s training and to estimate the optimal actions to take.
2. **State**: Each agent’s next action is determined by the decisions made in earlier phases. As a result, each agent’s state is composed of two components: a collection of previous actions and the current data to be processed. The number of observations in the database is used to determine the size of the state space $\mathcal {S}$.
3. **Action**: It is the assignment of each observation in the database’s decision-making behaviour. For instance, in a prediction task, it is the assignment of each object’s class.
4. **Reward**: Determining an appropriate reward function is critical. It enables each agent in $\mathcal {A}$ to learn more effectively. We used data that contained ground truth to create a reward for the agent’s actions. The following is the definition of the reward function:
  1 $$ \mathcal{R}\left({\mathcal{A}}_i,{\mathcal{U}}_i\right)=\left\{\begin{array}{c}1,\mathrm{if}\kern0.5em {\mathcal{A}}_i\left({\mathcal{U}}_i,{O}_j\right)=\mathcal{L}\left({O}_j\right);\\ {}0, otherwise,\kern5.75em \end{array}\right. $$
  where $\mathcal {A}_i(\mathcal {U}_j,O_j)$ shows the decision of the agent $\mathcal {A}_i$ whether the observation $O_j$ has correct action. Besides, the $\mathcal {L}(O_j)$ represents the ground-truth of the observation $O_j$.

So each agent $\mathcal {A}_i$ starts by scanning the observations of the $i^{th}$ smart sensor. It then computes the first and subsequent observations for the $i^{th}$ intelligent sensor. A reward function for this choice is constructed using the ground truth for the first observation. This procedure is performed for each observation of the $i^{th}$ intelligent sensor. This results in a collection of local choices, denoted $LD_i$, for each agent $\mathcal {A}_i$.

### Hyper-parameters optimization

We use an evolutionary-based mechanism for hyper-parameter optimization to ensure optimal performance. We proposed different algorithms based on a Memetic Algorithm (MA), and Bees Swarm Optimization (BSO). These evolutionary computation techniques were chosen for this purpose because of their well-known balance of intensification and diversification, both of which are critical in this setting. Next, a detailed explanation of MA and BSO is given for solving our hyper-parameters optimization problem.

Let $\mathcal {H}\mathcal {P}=\{\mathcal {H}\mathcal {P}_1, \mathcal {H}\mathcal {P}_2, \dots , \mathcal {H}\mathcal {P}_r\}$ be the set of the hyper-parameters in which *r* represents the number of hyper-parameters in the developed HI2D. For each $\mathcal {H}\mathcal {P}_i$, it represents a set of the potential values of the hyper-parameter. The configuration space $\mathcal {C}$ is then defined according to the set of all potential configurations in which each configuration is the vector by keeping the possible values of all the hyper-parameters in $\mathcal {H}\mathcal {P}$. For hyper-parameters optimization, it focuses on deriving the optimal configuration that can provide the best accuracy result. The configuration space’s size is determined by the number of all possible values for the hyper-parameters, as specified in Eq. 2.

2 $$\begin{array}{*{20}c} |\mathcal {C}|=\prod \limits _{i=1}^{r} |\mathcal {H}\mathcal {P}_i|\end{array}$$

The size of the configuration space is very huge, thus it takes a high computational cost to find the solutions. For example, if 1, 000 possible values are considered for epoch parameter, 100 possible value is considered for the error rate and 100 possible values is the number of the agents in the designed model, thus it requires 10 million configurations in the search space; it is not suitable by applying the exhaustive search methods. Evolutionary computation approaches are employed to address this problem. The main components of our solution are described as follows.

#### Population initialization

We attempt to distribute $pop\_size$ which is the initial population. This initial population should be evenly distributed in $\mathcal {C}$, which is the configuration space. This even distribution technique is allowed for the proper exploration of every of the many different configurations which tend to cover most regions within $\mathcal {C}$. We first must generate the initial population and maintain the diversity. This process itself is begun by randomly generating one individual that is represented by a single $\mathcal {C}$ configuration. Starting with this individual, we then can generate an additional $pop\_size-1$, where every new individual should be different than the individuals generated. We can make use of a distance measure between two back-to-back configurations to determine the dissimilarity using the individuals generated in those configurations. $\mathcal {P}$, shown as the initial population, should, in turn, be able to maximize the diversification function shown in Eq. 3.

3 $$\begin{array}{*{20}c} Diversify(\mathcal {P})=\sum \limits _{i=1}^{pop\_size} \sum \limits _{j=1}^{pop\_size} Distance(\mathcal {C}_i, \mathcal {C}_j),\end{array}$$

where $Distance(\mathcal {C}_i, \mathcal {C}_j)$ is the distance between the configurations of the $i^{th}$, and $j^{th}$ individuals, respectively.

#### Crossover

Each of the current population’s two individuals goes through the following steps to generate new offspring:

- From 1 to *r*, we generate a random series of crossing points, each of which we divide into two parts, the *left* and *right*.
- The left side of the original is duplicated on the left side of the first descendant and the right side of the original is duplicated on the right side of the second descendant.
- In the second generation, the left side of the second individual is inherited by the second generation, while the right side is inherited by the first generation.

#### Mutation

The process of mutation encourages the pursuit of diversity. We use a strategy where the value of a single parameter is randomly changed in each existing configuration. The mutation point is randomly generated and can have a value between 1 and *r* depending on the algorithm. At each iteration of the crossover operation, the crossover operator changes the value of the mutation point in the resulting offspring.

#### Local search

The local search operator is to find things in your environment by starting with an individual and working outward from there. When you change any of the hyperparameter values in the current configuration, a new area is created for you to work in. For the selected number of iterations, this process is repeated for each member of the population.

#### Fitness function

HI2D’s objective is to maximize infectious disease detection. Thus, we utilize the following function to assess individuals inside populations:

4 $$\begin{array}{*{20}c} Fitness(\mathcal {C}_i)=Detection_{HI2D}(\mathcal {C}_i)\end{array}$$

Note that,

- The configuration of the population’s $i^th$ individual is represented by $\mathcal {C}_i$ .
- $Detection_{HI2D}(\mathcal {C}_i)$ shows the detection ratio of the HI2D framework by using the $\mathcal {C}_i$.

Based on these operations, we proposed the following hyper-parameter optimization algorithms.

#### Memetic algorithm

To begin, the initial population size, defined as $pop\_size$, is generated randomly. Following that, each individual is constructed using population initialization. Following that, local search operators, mutation, and crossover are used to generate configurations from $\mathcal {C}$. To ensure a stable population size, each individual is evaluated using the fitness function, with an emphasis on retaining the first high-quality $pop\_size$ individuals. At this point, all others are removed. This process is then repeated indefinitely until the maximum number of iterations has been reached. Note that this algorithm is based on the genetic algorithm. The local search process is added in order to improve the exploration process.

#### Bees swarm optimization algorithm

Excellent features are discovered through the use of an initial bee that settles on a beneficial configuration. Using this initial configuration, a subset of configurations known as the *SearchArea* of the larger search space is determined. This is accomplished through the use of Eq. 3. Each bee uses a configuration from the *SearchArea* as a starting point. Once the local search processing is complete, each bee will communicate the configuration they believe is the best to all other neighbouring bees. This procedure is completed with the assistance of a table of *Dance*. During the subsequent iterations, one of the configurations from *Dance* will be designated as the reference configuration. To ensure that the specified number of cyclic iterations occurs, a taboo list of previous reference configurations is created. Each reference configuration is chosen based on its quality. We can also state that if and only if the actual swarm in its entirety is able to see the reference configurations are actually not gaining any improvement after some period, then a process for criteria diversification can be used in avoidance of becoming computationally trapped in local optimum which in reality provides no global benefit. Our taboo list can then be used for the generation of diversification criteria to be able to locate the reference configuration of the most distant past from our current stuck one. Our algorithm finally will terminate when and only when an optimal configuration is reached or in turn when some maximum number of iterations occurs.

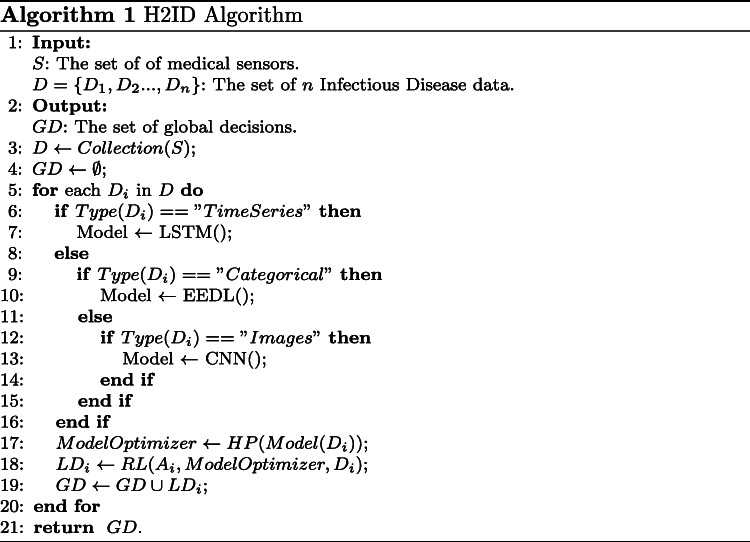



Algorithm 1 presents the pseudo-code of the H2ID algorithm. The algorithm starts by collecting the infectious disease data from medical sensors. For each data, we check its type, if it is time series, the long short term memory model is created, if it is categorical, the entity embedded deep learning is created, and if it is an image, the convolution neural network is created. The hyper-optimization process is then performed to identify the optimized model for the data at hand. The local decision of each agent is determined by applying the reinforcement learning process. This is applied to each agent in the system. The algorithm returns the global decision which will be the concatenation of local decisions of the agents.

## Performance evaluation

To validate the use of the proposed HI2D framework, extensive tests were undertaken on well-known medical databases. The experiments were conducted on a desktop computer equipped with an Intel *i7* processor and 16 GB of main memory. Python was used to implement all algorithms. The runtime evaluation is measured in mile-seconds, and the accuracy is measured by the ratio between the corrected detected infections and the number of all infections.

### Data description

We used three medical databases with different data representations raw data, time series, and images^1^. A detailed description of the data is given as follows,

1. **Respiratory Sound Data**: It is a raw data collection, includes annotated recordings of different patients. These recordings contain different respiratory cycles of crackles, wheezes and both crackles and wheezes. The data set includes both clean and noisy recordings of respiratory sounds that simulate real-world conditions [26].
2. **Real-time COVID-19 Data**: This is a COVID-19 time series documenting verified cases, reported deaths, and reported recoveries. Data were combined on a country-by-country basis. It is collected by Gaurav Dutta^2^. It contains time-series data on the number of people worldwide who have become infected with COVID-19, including i) confirmed, tested cases of coronavirus infection, ii) the number of people who died from coronavirus during their illness, and iii) the number of people who recovered.
3. **Corn Leaf Infection Data**: It is image collection to get an understanding of the cornfield and collect the corn leaf data that were partially infected by pests like Fall Armyworm. It is collected by Acharya^3^ to help the agriculture sector by making some systems that can help farmers using artificial intelligence.

**Fig. 2:**
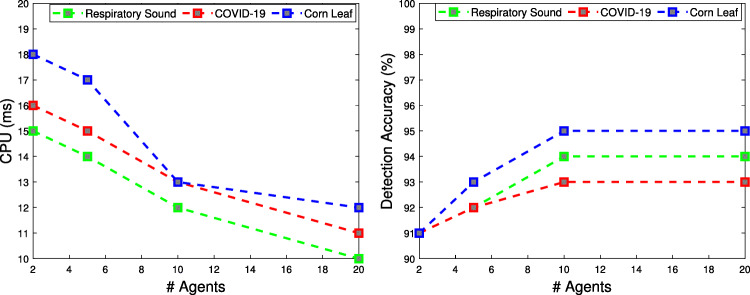
The running time and the detected accuracy of the proposed solution with different number of agents

**Fig. 3:**
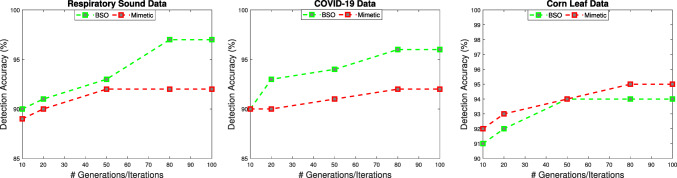
The detected accuracy of the proposed solution with different generations/iterations of the hyper-optimization strategy

**Fig. 4:**
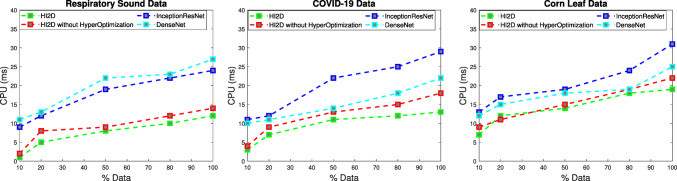
Runtime performance comparison of the H2ID and the existing infection disease intelligence detection

**Fig. 5:**
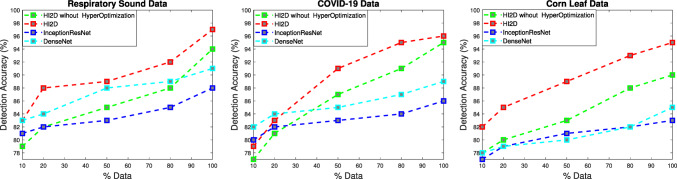
Accuracy performance comparison of the H2ID and the existing infection disease intelligence detection

### Parameters settings

The first tests have the objective to tune the parameters of the main components of the H2ID framework. Numerous experiments have been conducted with varying numbers of agents, and the number of generations/iterations, of the hyper-optimization algorithms. Fig. 2 shows the CPU time, and the detection accuracy of the H2ID framework by varying with the number of agents from 2 to 20 and using the three different data described above. The result reveals that by increasing the number of agents, we gain benefit from both runtime and detection accuracy. According to the figure, the number of agents should be set to 20. The result also reveals that by varying the number of generations of the Memetic algorithm, and the number of iterations, of the BSO algorithm from 10 to 100, an improvement in terms of accuracy is observed. According to Fig. 3, the hyper-parameter optimization algorithm should be set to BSO for Respiratory Sound, and COVID-19 data, and it should be set to a memetic algorithm for Corn Leaf data. Also, the number of generations/iterations should be set to 80. The next experiments seek to validate the applicability of the proposed H2ID framework for identifying disease infectious. To reach this conclusion, several comparisons have been made with the state-of-the-art algorithms (InceptionResNet [30], and DenseNet [28]). We first measure the runtime performance and then determine the quality performance. The results will be shown in the following.

### Runtime performance

Fig. 4 shows the runtime of the H2ID, InceptionResNet, and DenseNet using different data (Respiratory sound data, Covid-19, and Corn leaf data). By stretching the data used in the experiments, from $10\%$ to $100\%$, the computational time of algorithms increases from 10 ms to 30 ms. Counterpart, H2ID surpasses the baseline solutions in all cases. H2ID runtime does not exceed 15 ms for handling the entire data, however, the baseline solutions go up to 30 ms in processing the same portion of data. These results are mainly reached thanks to the efficient design of the H2ID, where distributed deep learning is performed efficiently. Indeed, communication is used among the different agents in the system to share the relevant outputs and then quickly converge to the optimal result.

### Detection accuracy

Fig. 5 shows the detection accuracy of the H2ID, InceptionResNet, and DenseNet using different data (Respiratory sound data, COVID-19, and Corn leaf data). While varying the data used as input, from $10\%$ to $100\%$, an improvement in terms of the detection accuracy of all algorithms is shown from 70 to 90. Also, H2ID outperforms the baseline solutions whatever the scenario running in the experiment. H2ID accuracy reaches $97\%$ in terms of corrected detection of infectious disease in processing the whole data, nevertheless, the accuracy of the two other solutions never reaches $93\%$ of corrected detection infectious disease. This success is explained by the coherent development of the proposed deep learning solutions in the H2ID, where different optimizations are developed including avoiding over-fitting with dropout layers, training a high number of layers using the batch normalization, and the accurate hyper-optimization setting by deploying evolutionary computation algorithms.

### H2ID Vs advanced infectious disease detection solutions

The final experiments aim to compare the performance of H2ID solutions with advanced infectious disease detection solutions. We used Xception [14] and SqueezeNet [2] for infectious disease detection. Table 1 shows both the runtime in milliseconds and the detection accuracy of H2ID, Xception, and SqueezeNet using different data (breath sound data, Covid-19, and corn leaf data). The results show the superiority of H2ID compared to the other two solutions in terms of detection accuracy in all cases. Moreover, it is very competitive with SqueezeNet in terms of runtime. This performance can be explained by the fact that SqueezeNet is an optimized deep learning architecture that is very well suited for use in mobile devices, but the quality of this architecture is not as good. In contrast, our solution performs well in terms of both runtime and accuracy.

**Table 1:** H2ID Vs. Advanced infectious disease detection solutions

Dataset	H2ID		Xception		SqueezeNet	
	CPU	Accuracy (%)	CPU	Accuracy (%)	CPU	Accuracy (%)
Respiratory Sound Data	12	95	17	91	13	88
Real-time COVID-19 Data	10	96	14	90	11	82
Corn Leaf Infection Data	17	94	21	93	16	85

## Future direction

This section presents the main outcomes of the application of the developed framework on medical data. We also suggest some future directions for improving the developed framework.

1. The efficient combination between the deep learning technologies, and the intelligent agents results in high accuracy performances. The runtime performance is still an issue for handling medical data in real-time. Combining metaheuristics and exact solutions [8] may be a good direction to improve both accuracy and runtime performance.
2. The proposed framework is successfully applied on detecting infectious diseases. It improved the existing baseline methods for infectious disease detection. It will be very interesting to see the results of the proposed framework on other medical domain applications such as brain tumour detection [35], surgery [25] and medical pattern recognition [18].
3. The proposed framework suffers from the output interpretation. It is based on black-box models that do not implicitly explain the execution path of the output generation. When deployed in healthcare settings, practitioners of machine learning systems need to understand how we arrived at a specific outcome and must trust it and its reliability. The emerging field of XAI (eXplainable Artificial Intelligence) addresses this need and offers several methods to provide some level of explanation to deep learning AI solutions. From a future perspective, we plan to integrate the XAI techniques in the proposed framework. This allows for giving a better interpretation of the developed framework.

## Conclusion

This paper proposed a new intelligent framework for infection disease detection by incorporating emerging intelligent components. Deep learning architectures such as convolution neural networks, long-short term memory networks, and entity embedding networks are adopted to learn both structured and unstructured medical data. A multi-agent system is also integrated into the suggested framework to increase intelligence, where each agent shares the learning results with the other agents in the system. Also, the evolutionary computation algorithms, such as memetic algorithm, and bees swarm optimization are conducted to smartly explore the configuration space of the possible combination of the hyper-parameter values. To demonstrate the applicability of the proposed framework, intensive experiments have been carried out on medical data. The results plainly show the effectiveness of our methodology against the baseline solutions.

## Footnotes

1 https://www.kaggle.com/

2 https://www.kaggle.com/gauravduttakiit/covid-19

3 Acharya, R. (October 2020) Corn Leaf Infection Dataset, Version 1. Retrieved October 2020 from https://www.kaggle.com/qramkrishna/corn-leaf-infection-dataset
